# Clinical performance study of a fecal bacterial signature test for colorectal cancer screening

**DOI:** 10.1371/journal.pone.0293678

**Published:** 2023-11-22

**Authors:** Marta Malagón, Elizabeth Alwers, Lia Oliver, Sara Ramió-Pujol, Mireia Sánchez-Vizcaino, Joan Amoedo, Salomé de Cambra, Mariona Serra-Pagès, Antoni Castells, Xavier Aldeguer, Jesús Garcia-Gil, Hermann Brenner

**Affiliations:** 1 GoodGut SLU, Girona, Spain; 2 Division of Clinical Epidemiology and Aging Research, German Cancer Research Center (DKFZ), Heidelberg, Germany; 3 HIPRA Human Health, Amer, Spain; 4 Hospital Clínic de Barcelona, Institut d’Investigacions Biomèdiques August Pi i Sunyer (IDIBAPS), CIBERehd, Universitat de Barcelona, Barcelona, Spain; 5 Institut d’Investigació Biomèdica de Girona-IDIBGI, Salt, Spain; 6 Departament de Biologia, Universitat de Girona, Girona, Spain; 7 Division of Preventive Oncology, National Center for Tumor Diseases (NCT) and German Cancer Research Center (DKFZ), Heidelberg, Germany; 8 German Cancer Consortium (DKTK), German Cancer Research Center (DKFZ), Heidelberg, Germany; University of Minnesota Twin Cities, UNITED STATES

## Abstract

The fecal immunochemical test (FIT) is the most widely used test for colorectal cancer (CRC) screening. RAID-CRC Screen is a new non-invasive test based on fecal bacterial markers, developed to complement FIT by increasing its specificity. The test was previously clinically evaluated in FIT-positive patients (>20 μg of hemoglobin/g of feces, “FIT20”), in which it reduced the proportion of false positive results by 16.3% while maintaining most of FIT20’s sensitivity. The aim of this study was to compare the sensitivity and specificity of a CRC screening program using RAID-CRC Screen in addition to FIT20 as a triage test in a European screening population undergoing screening colonoscopy with a CRC screening program with FIT20 alone in the same cohort. A cohort of 2481 subjects aged > 55 years from the German screening colonoscopy program was included. The colonoscopy findings were used as the gold standard in calculating the diagnostic capacity of the tests and included 15 CRC and 257 advanced neoplasia cases. RAID-CRC Screen added to FIT20 provided the same sensitivity as FIT20 alone (66.7%) in detecting CRC and a significantly higher specificity (97.0% vs. 96.1%, p<0.0001). The positive predictive value was 11.9% when using RAID-CRC Screen and 9.5% with FIT20 alone, and the negative predictive value was 99.8% in the two scenarios. For advanced neoplasia detection, the use of RAID-CRC Screen yielded significantly lower sensitivity than with FIT20 alone (17.5% vs. 21.8%, p = 0.0009), and the overall specificity was significantly higher when using RAID-CRC Screen compared with FIT20 alone (98.2% vs. 97.8%, p = 0.0039). Our findings confirm the results obtained in previous clinical studies in a CRC screening setting, showing the potential of RAID-CRC Screen to increase the overall specificity of FIT-based screening.

## Introduction

Colorectal cancer (CRC) is the third most-frequently diagnosed cancer and the second in mortality worldwide, accounting for more than 935,000 deaths in 2020 [1]. CRC is susceptible to screening as it represents a significant health burden, presents a step-wise evolution providing a time window opportunity, and has diagnostic tools for its early detection, while treatment outcomes are improved when detected early [2]. CRC screening programs can effectively reduce incidence and mortality rates in the target population [3].

Most European countries offer CRC screening to average-risk populations from the age of 50 years, based on a two-step approach in which non-invasive tools are used as a triage test to determine who should proceed to a confirmatory colonoscopy [4]. Widely adopted globally, the fecal immunochemical test (FIT) quantifies human hemoglobin in feces [4, 5]. The main advantage of FIT is that it is quantitative, and the cut-off for defining a positive result can be adjusted according to endoscopic capacity in the healthcare system [6].

The use of lower cut-off values of FIT enables a higher detection rate of colorectal tumoral lesions. However, this comes at the expense of reduced specificity, generating a higher false-positive rate and, therefore, increasing the number of unnecessary colonoscopies which, in turn, may result in potential adverse events and additional healthcare costs. An alternative strategy might be to increase the effectiveness and efficiency of population-based screening programs. To this end, a new tool called RAID-CRC Screen was developed to improve the specificity of FIT, thereby reducing the false-positive rate as part of a three-step approach to the CRC screening program [7].

RAID-CRC Screen is a non-invasive tool for CRC screening based on a specific fecal bacterial signature that, used in addition to a positive FIT result, increases the overall specificity and positive predictive value (PPV) of both CRC and advanced neoplasia (advanced adenoma and/or CRC) detection by selecting for colonoscopy only patients with a positive fecal bacterial signature in addition to a positive FIT result [7]. The fecal bacterial signature includes six biomarkers: *Gemella morbillorum* and *Bacteroides fragilis* as opportunistic pathogens; *Faecalibacterium prausnitzii* [8], B46 (mixture of bacterial DNA sequences with a best BLAST match for *Subdoligranulum variabile*), and B48 (mixture of bacterial DNA sequences with a best BLAST match for *Ruminococcus*, *Roseburia*, and *Coprococcus*) as butyrate-producing bacteria [9]; and Eubacteria as the total bacterial load [10].

In a first proof-of-concept study (RAID-CRC 20202015) in which 172 patients with a positive FIT were enrolled (cut-off, 20 μg hemoglobin/g of feces; FIT20), adding RAID-CRC Screen reduced the proportion of false positive FIT20 results by 34% (32/94), while maintaining 100% and 94% of FIT20’s sensitivity for CRC and advanced neoplasia (AN), respectively [7]. A first clinical performance study in 327 FIT20-positive patients (GG-RAIDCRC-1002) showed similar results: false positive results were reduced by 16.3% (30/184), and FIT20’s sensitivity for CRC and AN was maintained at 94.7% and 83.9%, respectively [7]. This preliminary data showed the potential of the tool to improve the clinical utility of FIT, suggesting a step towards a more accurate and affordable diagnosis for screening programs. However, the study population consisted of FIT20 positive subjects only and diagnostic performance could be evaluated in this subgroup only, thus quantifying sensitivity compared with FIT20. Accordingly, evaluation of its effectiveness in unselected CRC screening participants was required.

The aim of this study was to compare the sensitivity and specificity of a CRC screening program using RAID-CRC Screen in addition to FIT20 as a triage test in a European screening program undergoing screening colonoscopy with a CRC screening program with FIT20 alone in the same cohort.

## Materials and methods

### Study design and population

The analysis is based on data from the BLITZ study, a large ongoing study conducted in the context of the German screening colonoscopy program in which blood and stool samples are collected from average-risk persons before a routine screening colonoscopy. The German screening colonoscopy program offers colonoscopy as a primary screening examination for men and women aged ≥ 55 years (in 2019 the starting age for men was lowered to 50 years). Screening colonoscopies are mostly conducted in gastroenterology practices, and the program has implemented rigorous quality control measures. The study was approved by the ethics committee of the Heidelberg Medical Faculty of Heidelberg University. Written informed consent including usage of stored samples for future studies was obtained from each participant. Further details of the BLITZ study have been previously reported [11–14].

Fecal samples collected before bowel cleansing from participants aged ≥ 55 years recruited in 2012–2016 were retrospectively selected for inclusion in this analysis. We excluded samples from participants who had a personal history of CRC, colorectal adenomas, colorectal polyposis, or inflammatory bowel disease, a positive fecal occult blood test in the six months before fecal sample collection, had undergone colonoscopy or flexible sigmoidoscopy in the five years prior to study recruitment, had received antibiotic treatment within one month or chemotherapy and/or radiotherapy within 6 months of fecal sample collection, had severe comorbidities, or other diseases and/or surgery that compromised the transit of the digestive system, or pregnancy at the time of fecal sample collection.

A minimum of 248 fecal samples from subjects with advanced colorectal neoplasm (advanced adenoma, advanced serrated lesions, or CRC; true disease status defined by colonoscopy followed by histopathological examination), and 1507 fecal samples from participants with negative colonoscopy (normal or non-advanced adenoma) were required to achieve 80% power to demonstrate both non-inferiority in sensitivity and superiority in specificity of the RAID-CRC Screen approach *vs*. FIT20 alone. Two one-sided McNemar tests with a significance level of 0.05 (estimated proportions of discordant pairs of 10%) was used. With an estimated 10% advanced neoplasm prevalence, approximately 2480 samples were included retrospectively to achieve a sufficient number of positive colonoscopies.

### Colonoscopy findings

Colonoscopy and histology reports were reviewed by trained investigators who were blinded to the fecal testing results. Each participant was classified according to the most advanced finding at colonoscopy. Adenomas were defined as advanced if they matched any of the following features: size ≥1 cm, tubulovillous or villous architecture, or high-grade dysplasia. Advanced Neoplasia (AN) include CRC, advanced adenoma, pTis neoplasia and adenocarcinoma *in situ*, sessile serrated adenoma ≥1 cm, or any other finding with high-grade dysplasia.

### Fecal sample collection and analysis

Stool samples were collected at home using sterile feces container before study participants started bowel preparation for the colonoscopy. Samples were stored by the participant in the freezer (or refrigerator, if freezing was not possible) and brought to the gastroenterology practice in a temperature isolated bag on the day of the colonoscopy. Thereafter, the samples were stored at -20ºC, sent in a cooling chain to a central laboratory, and finally stored at -70ºC in the German Cancer Research Center (DKFZ).

For this analysis, 100 mg aliquots obtained from the deep-frozen stool samples at the DKFZ biobank were transported in frozen conditions to GoodGut (Girona, Spain).

Fecal samples were thawed to collect samples with the FIT collector for the FIT analysis and to extract DNA. FIT was performed using OC-Sensor IO (Eiken Chemical Co., Tokyo, Japan) 48 hours after sample thawing. DNA was extracted using the DNeasy Powersoil Pro kit (Qiagen, Hilden, Germany) according to the manufacturer’s instructions and eluted to 100 μl final volume and stored at -20ºC until use.

### qPCR assay for RAID-CRC Screen biomarkers

The specific bacterial sequences targeted were those included in RAID-CRC Screen: Eubacteria (EUB), B46, B48, *F*. *prausnitzii* (FPRA), *G*. *morbillorum* (GMLL), and *B*. *fragilis* (BCTF) [7]. Biomarkers were quantified by preparing two multiplex qPCRs (multiplex 1, EUB-B46-GMLL; multiplex 2, B48-BCTF) and a singleplex reaction (FPRA) using GoTaq® qPCR Probe Master Mix (Promega, Madison, USA). The sequences of the forward and reverse primers and probes are described in Table 1. Each reaction consisted of 10 μl containing 1× GoTaq® qPCR Probe Master Mix, between 50 nM and 300 nM of each primer and/or probe, and the DNA template. The species-specific primers were purchased from Macrogen (Macrogen, Seoul, South Korea). All samples were amplified in duplicate. A no-template control reaction and a standard curve (sequential dilutions of known concentrations) were included in each qPCR run to check the efficiency of amplification. All qPCRs were run on an AriaDx Real-time PCR System (Agilent Technologies, Santa Clara, USA). The thermal profile used consisted of an initial cycle of 1 minute at 95ºC, 40 cycles of 15 seconds at 95ºC and 30 seconds at 60ºC. Data were collected and analyzed using Aria Software version 1.71 (Agilent Technologies, Santa Clara, USA).

**Table 1:** Forward and reverse primers and probe sequences included in RAID-CRC Screen. EUB, Eubacteria; GMLL, *G*. *morbillorum*; BCTF, *B*. *fragilis*; FPRA, *F*. *prausnitzii*; F, Forward primer; R, Reverse primer; PR, probe. Fluorochromes and quenchers are also shown in the probes’ sequences.

Target	Primers / Probe	Sequence 5’ → 3’	Concentration (nM)	Ref
EUB	EUB2_F	CGG TGA ATA CGT TCC CGG	50	[15]
	EUB2_R	TAC GGC TAC CTT GTT ACG ACT T		
	EUB2_PR	FAM-CTT GTA CAC ACC GCC CGT C-BHQ1	60	
B46	B46_F	TCC ACG TAA GTC ACA AGC G	300	[16]
	B46_R	CGC CTA CCT GTG CAC TAC TC		
	B46_PR	HEX-ATT ACT GGG TGT AAA GGG AGC GCA-BHQ1	100	
B48	B48_F	GTA CGG GGA GCA GCA GTG	300	[16]
	B48_R	GAC ACT CTA GAT GCA CAG TTT CC		
	B48_PR	HEX-CGC GTG AGC GAA GAA GTA TTT CGG T-BHQ1	100	
GMLL	GMLL_F	AAG AGT TCC AAG GCG TTC TC	300	[17]
	GMLL_R	CCA TTT CAA GAT CCG CTT TCT ATT T		
	GMLL_PR	ROX-CCA TCA AAT CTT TCT TTC TCT TCA TCT GGG-BHQ2	150	
BCTF	BCTF_F	TGA AAG CGT GCT CTT ACT ATT G	250	[17]
	BCFT_R	TAT TGG CTG TTG TGC TTT GT		
	BCTF_PR	CY5-ACG TTT GCC CGG AGT GAC TTC TAT-BHQ2	200	
FPRA	FPRA_F	TGT AAA CTC CTG TTG TTG AGG AAG ATA A	300	[8]
	FPRA_R	GCG CTC CCT TTA CAC CCA		
	FPRA_PR	FAM-CAA GGA AGT GAC GGC TAA CTA CGT GCC AG-BHQ1	250	

* Each reaction consisted of 10 μl containing 1× GoTaq® qPCR Probe Master Mix, primer and/or probe, and the DNA template.

In the qualitative analysis, the absence of a biomarker was defined as a C_t_ value obtained outside the dynamic range, i.e. the interval of relative abundance in which a given bacterial marker can exist.

Investigators analyzing the samples were blinded to the participants’ diagnoses.

### RAID-CRC Screen

The RAID-CRC Screen algorithm had previously been defined in a proof-of-concept study (study code RAID-CRC 20202015) in 172 subjects who participated in the Catalan CRC screening program and had a FIT- positive result [7]. Specifically, a combination of bacterial markers that enable discrimination of individuals with AN from those with normal colonoscopy or non-advanced adenomas was selected. The design of the algorithm consisted of an initial training iteration with 100 random partitions of 70% of the dataset using machine learning. The resulting algorithm was further validated using the remaining 30% to check for reproducibility. The RAID-CRC Screen algorithm consists of a decision-tree that includes the relative abundance of five bacterial markers. RAID-CRC Screen is applied as a triage test only to subjects who have tested FIT≥ 100 ng/ml (20 μg Hb/g feces) positive. The algorithm included in RAID-CRC Screen uses five ratios, since each bacterial marker is normalized using Eubacteria to obtain the relative abundance of each biomarker. Data normalization is critical to control qPCR‐associated variables in order to differentiate true biological changes from experimentally-induced variation.

### Statistical analysis

Sensitivity, specificity, overall accuracy, positivity rate, positive and negative likelihood ratios, and positive and negative predictive values of a CRC screening program using RAID-CRC Screen in addition to FIT as a triage test were compared with those of a CRC screening program using FIT alone, using in both cases the FIT cut-off value recommended by the manufacturer (100 ng/ml, which corresponds to 20 μg Hb/g feces). The diagnostic accuracy was determined using the results of the screening colonoscopy as the gold standard. Endpoints evaluated were (i) detection of AN; and (ii) detection of CRC.

Differences in sensitivity, specificity, accuracy, and the positivity rate between the use or not of RAID-CRC Screen together with FIT as a triage test were evaluated for statistical significance by the McNemar test; statistical significance was defined as a p-value <0.05 in a two-sided test. Differences in likelihood ratios were evaluated using a regression model [18] with a 0.05 significance level. Differences in positive and negative predictive values were evaluated using a generalized score statistic for paired tests [19]. All significance tests were performed using the DTComPair package [20] on R software (version 3.6.1) [21].

To evaluate the concordance of our results with those of other studies in independent cohorts that were based on FIT positive participants only [7], we additionally derived measures of diagnostic performance that would be obtained in the subgroup of FIT20 positive patients (N = 105) only. In this subgroup, the sensitivity indicates the proportion of FIT20 true positives that RAID-CRC Screen will also identify as positive, and the specificity indicates the proportion of FIT20 false positives that RAID-CRC Screen will correctly identify as true negatives.

## Results

In the 2481 screening colonoscopy participants (mean age 62.8 years, 49% male), advanced neoplasms were detected in 257 (10.3%) participants, and 84 (3.4%) were classified as positive when using RAID-CRC Screen in addition to FIT20 as a triage test. Table 2 presents the most advanced finding of the screening colonoscopy among all participants included and among those classified as positive for AN by RAID-CRC Screen in FIT20-positive patients.

**Table 2:** Colonoscopy findings in the study population and classified positive for advanced neoplasia by RAID-CRC Screen in addition to FIT20.

Diagnostic	Participants (N = 2481)	Percentage (%)	RAID-CRC Screen Positive (n = 84)	Percentage (%)
Colorectal cancer	15	0.6	10	66.7
Advanced adenoma	211	8.5	35	16.6
Serrated polyp/adenoma (≥1 cm)	31	1.2	0	0.0
Non-advanced adenoma	403	16.2	8	2.0
Serrated polyp/adenoma (<1 cm)	42	1.7	1	2.4
Hyperplastic polyp	248	10.0	4	1.6
Non-specified polyp	73	2.9	3	4.1
Normal colonoscopy	1458	58.8	23	1.6
Total	2481	100	84	3.4

When RAID-CRC Screen was applied in addition to FIT20 it yielded an overall sensitivity of 17.5% and specificity of 98.2% for the detection of AN and a sensitivity of 66.7% and specificity of 97% for CRC detection.

For the detection of AN, RAID-CRC Screen added to FIT20 had significantly higher specificity than FIT20 alone (98.2% vs 97.8%, p = 0.004) and significantly-lower sensitivity (17.5% vs. 21.8%, p = 0.001). No significant differences were observed regarding the PPV or positive likelihood ratio (LR) and only marginal differences were found for the NPV and negative LR (Table 3).

**Table 3:** Diagnostic performance of RAID-CRC Screen added to FIT20 vs FIT20 alone for detection of advanced neoplasia.

	RAID-CRC Screen		FIT20 alone		
Parameter	n/N	(%)	n/N	(%)	p-value^1^
Sensitivity	45/257	17.5	56/257	21.8	0.0009
Specificity	2185/2224	98.2	2175/2224	97.8	0.0039
PPV	45/84	53.6	56/105	53.3	0.9259
NPV	2185/2397	91.2	2175/2376	91.5	0.0025
Accuracy	2230/2481	89.9	2231/2481	89.9	-
Positive LR	-	9.99	-	9.89	0.9261
Negative LR	-	0.84	-	0.80	0.0026

^1^p-values for comparison of RAID-CRC Screen in addition to FIT20 with FIT20 alone. PPV: Positive predictive value; NPV: Negative predictive value; LR: Likelihood Ratio.

For the detection of CRC, RAID-CRC Screen combined with FIT20 had significantly-higher specificity than FIT20 alone (97% vs 96.1%, p<0.0001) and equal sensitivity (66.7%). The PPV and positive LR were marginally higher for the RAID-CRC Screen and FIT20 combination, whereas no differences were observed for the NPV and negative LR (Table 4).

**Table 4:** Diagnostic performance of RAID-CRC Screen in addition to FIT20 vs FIT20 alone for detection of CRC.

	RAID-CRC Screen		FIT20 alone		
Parameter	n/N	(%)	n/N	(%)	p-value^1^
Sensitivity	10/15	66.7	10/15	66.7	-
Specificity	2392/2466	97.0	2371/2466	96.1	<0.0001
PPV	10/84	11.9	10/105	9.5	NA
NPV	2392/2397	99.8	2371/2376	99.8	NA
Accuracy	2402/2481	96.8	2381/2481	96.0	<0.0001
Positive LR	-	22.22	-	17.31	NA
Negative LR	-	0.34	-	0.35	NA

^1^p-values for comparison of RAID-CRC Screen in addition to FIT20 with FIT20 alone. PPV: Positive predictive value; NPV: Negative predictive value; LR: Likelihood Ratio. NA: No reliable estimate possible due to low CRC case numbers

We conducted a *post-hoc* analysis considering only FIT20 positive patients (N = 105). In the subgroup of FIT20 positive patients (N = 105), 45 out of 56 participants with AN and 10 out of 10 participants with CRC also had a positive RAID-CRC Screen (Tables 3 and 4), which corresponds to sensitivities of 80% and 100%, respectively. The proportions of FIT20 false positives correctly identified as true negatives were 10/49 (20%) for AN and 21/95 (22%) for CRC.

## Discussion

CRC screening programs focused on the average-risk population have been recommended, as they have been shown to reduce CRC incidence and mortality [22]. Early detection and removal of precursor lesions halt malignant transformation, thus preventing CRC and improving treatment outcomes and prognosis. At present, to maximize the impact of secondary prevention strategies, most national health systems aligned with the agenda of the European Plan to Fight Cancer have committed to set out plans to improve coverage and participation rates of screening programs, such as reducing the age to enroll in the program or lowering the FIT cut off. These measures will undoubtedly lead to more polyps/adenomas and cancers being detected, albeit at the same time implying more people being referred for colonoscopy. Hence, these objectives would lead to a greater demand for endoscopic resources, which currently limits their viability. This shows the need to introduce strategies to ensure that screening programs are capable of overcoming rate-limiting factors.

One potential strategy to create the capacity to cope with the future demand is increasing the diagnostic specificity of current CRC detection techniques. This will permit more accurate referrals for colonoscopy. Improving diagnostic accuracy also makes it possible to address other current needs and challenges of screening programs. Unscreened patients report that the main barriers to participation in CRC screening programs are fear related to the procedure, sedation, logistics, and discomfort with the procedure or preparation [23]. Patients report that colonoscopies negatively impact their quality of life, with high levels of pain reported in association with post-colonoscopy abdominal complaints [24, 25]. Likewise, they entail an increase in the care burden and contribute to the saturation of the health system due to the increase in waiting lists, intensifying the pressure of a system already in tension due to the limited availability of endoscopic resources. Taken together these factors entail an economic cost which, if optimized, would permit alleviation of the budgetary pressure suffered by the health system. In this context, we evaluated the capacity of the RAID-CRC Screen triage test to reduce the false-positivity rate of FIT20 alone by using it as an additional positivity criterion in a German cohort of patients at average risk for CRC.

Adding the bacterial signature of RAID-CRC Screen as an additional positivity criterion to FIT20 in CRC screening programs resulted in a significant reduction in false positive rates of 20% and 22% for the AN and CRC endpoints, respectively. For the CRC endpoint this was achieved while maintaining the same level of sensitivity as that obtained using FIT20 alone, but the sensitivity of detecting advanced adenomas, and hence the overall sensitivity in detecting AN, was significantly reduced (from 21.8% to 17.5%, a reduction of approximately 20%). These results are in line with and confirm previous results obtained in a clinical study which was restricted to participants preselected by a positive FIT in clinical practice [7].

The gain in specificity and reduction in unnecessary colonoscopies of approximately 20% estimated for the additional use of RAID-CRC Screen in our study, should be weighed against the loss in sensitivity of approximately 20% in detecting advanced neoplasms. An additional consideration is the time course of colorectal carcinogenesis. The development from advanced neoplasm to CRC typically takes many years, and a substantial proportion of advanced neoplasms will not develop into clinically manifest CRC during screenees’ lifetime [26]. The lower sensitivity for advanced neoplasm detection (in contrast to a lower sensitivity for CRC detection) might therefore be of limited concern (or might potentially even be advantageous) as it would preferentially concern early advanced neoplasms (or those that would never evolve to CRC during the patient’s lifetime). Most FIT-based screening programs offer annual or biennial screening, which implies that there is a good chance of early detection of advanced neoplasms that progress towards CRC, which may reduce concerns about the potential loss of sensitivity in detecting all advanced neoplasms found. Likewise, repeat screening rounds are expected to be accompanied by gradually decreasing prevalence of advanced neoplasms and further decreases in the positive predictive values of FIT-based CRC screening, which would increase the need for enhanced specificity.

As stated by the Colorectal Cancer Screening Committee of the World Endoscopy Organization, when considering the introduction of a new test and its clinical accuracy in the screening context, the effect on other variables in the screening pathway should be considered [27]. In particular, the feasibility of implementation with minimal changes in the clinical pathway may be important. For the RAID-CRC Screen, sample handling was adapted to the current pathway of the Catalan CRC screening program, optimizing sample collection in the FIT collectors so that both FIT analysis and bacterial DNA extraction can be performed using the same container [7, 17]. With respect to the workload, an automated DNA extraction procedure has been optimized, and the quantification of the bacterial markers included in the algorithm has been multiplexed for parallel quantification, thereby minimizing the increase in time and resources. A particularly relevant aspect with respect to resources and costs is that the bacterial signature would be selectively determined in FIT20 positive individuals only, in whom savings due to avoiding unnecessary colonoscopies would be the goal. One limitation of the study is that it does not replicate the real-world performance of FIT, which is done on fresh stool. However, previous assays in which the qPCR results obtained from fresh and frozen fecal samples collected with the FIT collector were compared, showed reproducibility.

While the bacterial signature of RAID-CRC Screen was shown to increase specificity in our study, its use should be further compared with alternative approaches to increase specificity, such as an increase or decrease in FIT positivity cut-offs [28, 29] or the combination of FIT with other non-invasive tests [30]. Given, however, that at the moment the new test is proposed as a triage test, it would seem more promising to test it when using a lower (not a higher) FIT cut-off. In such a scenario, the high specificity of the new test and the high sensitivity for CRC would allow the detection rate to be increased without increasing the number of colonoscopies. Using lower cut-off levels would also permit more lesions to be detected with a more precise estimate of sensitivity and specificity of the test among FIT positive subjects. Alternative options to be considered in FIT-based screening programs might include extending FIT screening intervals in subjects with low FIT values in preceding screening rounds [31], which would also reduce false positive rates and increase sensitivity and positive predictive values through increased prevalence rates of advanced neoplasms in subsequent screening rounds. Future research should aim for a more comprehensive, comparative evaluation of different approaches (or their combination) to enhance current FIT-based screening and include both head-to-head comparisons of different screening strategies and thorough modeling of their long-term impact and cost-effectiveness.

One of the RAID-CRC Screen medical device characteristics is that it is based on the fecal bacterial signature, which suggests that this signature could not be a universal panel since the dietary products and the associated eating habits and geographical provenance of individuals all have an influence on gut microbiota diversity [32, 33]. Although similar performance of the test in a Spanish population [7] and this German population, two populations with quite divergent dietary habits, suggests this to be of limited concern, further research should address performance across populations with a broad range of dietary habits.

The study has strengths and weakness. Major strengths are the large overall sample size in an average-risk screening population, and the availability of screening colonoscopy results for all participants. However, despite the large overall size, the number of participants with CRC was still rather small (n = 15) which reflects the low prevalence of CRC in screening settings. Estimates of sensitivity for CRC detection and the impact of adding RAID-CRC Screen to FIT20 on that sensitivity, therefore, need to be interpreted with caution. Likewise, the results were restricted to the use of RAID-CRC Screen in the FIT20 cut-off of one specific FIT brand only. Future studies should assess the performance of the combination of RAID-CRC Screen with other FIT brands and other positivity thresholds. In particular, based on the data obtained it would be promising to explore the combination when the FIT cut-off is lowered. In such a scenario, it is expected that the increased sensitivity provided by FIT and the potential higher specificity achieved when adding RAID-CRC Screen as a triage test would lead to higher detection rates with greater precision, thus requiring fewer colonoscopies than when using FIT alone. Even though the results were highly consistent with those from a preliminary Spanish clinical study [7], further replication in other, larger studies is highly desirable. Further studies should also aim to evaluate the performance of the test in other populations from other countries.

## Conclusions

Our results suggest that a three-step approach using the combination of FIT20 and RAID-CRC Screen as a triage test to select subjects for colonoscopy may potentially avoid a significant number of unnecessary colonoscopies compared with a two step approach where FIT is used alone. Further research should address the effectiveness and cost-effectiveness of using RAID-CRC Screen along with FIT-based screening compared with existing and alternative screening options in order to best define its potential use for enhanced CRC screening.

## Supporting information

S1 Checklist STROBE statement—checklist of items that should be included in reports of observational studies. (DOCX)
